# Neurofilament light chain and glial fibrillary acid protein levels are elevated in post-mild COVID-19 or asymptomatic SARS-CoV-2 cases

**DOI:** 10.1038/s41598-024-57093-z

**Published:** 2024-03-18

**Authors:** Domenico Plantone, Angela Stufano, Delia Righi, Sara Locci, Ivo Iavicoli, Piero Lovreglio, Nicola De Stefano

**Affiliations:** 1 Department of Medicine, Surgery, Neuroscience University of Siena, Siena, Italy; 2 Department of Interdisciplinary Medicine, University of Bari Aldo Moro, Bari, Italy; 3 Department of Public Health, University of Naples Federico II, Naples, Italy

**Keywords:** COVID-19, SARS-CoV-2, Neurofilament light chain, Glial fibrillary acidic protein, Cognition, Occupational risk, Health occupations, Neurology

## Abstract

Given the huge impact of the COVID-19 pandemic, it appears of paramount importance to assess the cognitive effects on the population returning to work after COVID-19 resolution. Serum levels of neurofilament light chain (sNfL) and glial fibrillary acidic protein (sGFAP) represent promising biomarkers of neuro-axonal damage and astrocytic activation. In this cohort study, we explored the association between sNfL and sGFAP concentrations and cognitive performance in a group of 147 adult workers with a previous asymptomatic SARS-CoV-2 infection or mild COVID-19, one week and, in 49 of them, ten months after SARS-Cov2 negativization and compared them to a group of 82 age and BMI-matched healthy controls (HCs). sNfL and sGFAP concentrations were assessed using SimoaTM assay Neurology 2-Plex B Kit. COVID-19 patients were interviewed one-on-one by trained physicians and had to complete a list of questionnaires, including the Cognitive Failure Questionnaire (CFQ). At the first assessment (T0), sNfL and sGFAP levels were significantly higher in COVID-19 patients than in HCs (p < 0.001 for both). The eleven COVID-19 patients with cognitive impairment had significantly higher levels of sNfL and sGFAP than the others (p = 0.005 for both). At the subsequent follow-up (T1), sNfL and sGFAP levels showed a significant decrease (median sNfL 18.3 pg/mL; median sGFAP 77.2 pg/mL), although they were still higher than HCs (median sNfL 7.2 pg/mL, median sGFAP 63.5 pg/mL). Our results suggest an ongoing damage involving neurons and astrocytes after SARS-Cov2 negativization, which reduce after ten months even if still evident compared to HCs.

## Introduction

Neurological manifestations have been associated with Coronavirus disease (COVID-19), both in the acute phase and the period following the infection resolution^1,2^ These manifestations have been characterized in detail and, together with the evidence from neuropathological studies, unequivocally demonstrate the involvement of the central nervous system (CNS) in COVID-19 patients^3^. However, the exact pathogenesis of CNS damage largely remains speculative, with direct viral CNS invasion and indirect inflammatory-mediated CNS injury as the main mechanisms hypothesized.

To investigate the CNS damage induced by SARS-Cov2 infection, neurofilament light chain (NfL) and glial fibrillary acidic protein (GFAP) have been proposed as two promising cross-disease biomarkers of neuronal and glial degeneration, respectively^4,5^. The discovery of digital ultrasensitive immunoassay methods has also allowed the measurement of these biomarkers in serum, where they were previously undetectable^6^. NfL (sNfL) and GFAP (sGFAP) levels in serum were found to be increased in hospitalized^7–10^ and non-hospitalized^11^ COVID-19 patients during the acute phase of the disease, independently of the presence of neurological symptoms. The elevation of these biomarkers in the period following COVID-19 resolution, however, has not been deeply investigated yet, although it should be considered a topic of great interest given the possible long-term implications of COVID-19 and the enormous spread of the disease^8^.

Research on mild COVID-19 cases indicates a concerning association with cognitive deficits in the general population, regardless of COVID clinical course and severity^12^and with no association with any blood inflammatory marker^13^Particularly, a recent study suggests that even in mild cases COVID-19 infection promote systemic endothelial dysfunction, impair the homeostatic mechanism of neurovascular coupling and promote white matter damage, contributing to the progression of mild cognitive impairment^14^. In another study, post-COVID cognitive dysfunction has been described in 43% of patients and associated with younger age^15^ In fact, this cognitive decline is not limited to older adults, as evidenced by another study that documented executive functioning deficits in young and middle-aged adults who were never hospitalized during acute COVID-19^16^.

Overall, these studies underscore the need for comprehensive monitoring and further research into the cognitive consequences of even mild COVID-19 or asymptomatic SARS-CoV-2 infection in the broader population. Furthermore, the potential impact of even mild neurological damage caused by the SARS-CoV-2 infection, and the consequent cognitive alterations has been little studied in occupational settings, with only a few studies exclusively focused on healthcare workers^17,18^ Given the huge impact of the COVID-19 pandemic, with a large number of infected individuals, it appears of paramount importance to monitor the cognitive effects on the population returning to work after COVID-19 resolution, especially in occupational contexts where such alterations could have a greater impact on the employees' well-being.

To explore the association between the levels of CNS damage biomarkers sNfL and sGFAP and cognitive performance, a group of adult workers with a previous asymptomatic SARS-CoV-2 infection or mild COVID-19 was investigated in the first week and subsequently ten months after test negativization.

## Methods

### Study design and population

This study was conducted between January 2022 and May 2023 during the prevalent circulation of the Omicron variant in Italy^19^. We enrolled a cohort of workers from the University of Bari (Apulia, Italy), who requested to be assessed by the University Occupational Health Unit physicians, following an asymptomatic SARS-CoV-2 infection or a mild COVID-19 diagnosed by molecular test (COVID-19 patients), according to the internal procedure for returning to work. The calculation of the minimum sample size was performed a priori considering the prevalence of long COVID in a previous study in a similar population^20^ and taking into account the results of the Global Burden of Disease analysis^21^ accepting a 2% margin of error and a 95% confidence interval. Mild COVID-19 individuals were those who presented any of the several signs and symptoms of COVID-19 (e.g. fever, sore throat, cough, malaise, migraine, muscular pain, vomiting, nausea, diarrhea, and loss of taste and smell), but without shortness of breath, dyspnoea or abnormal chest images^20^. Asymptomatic infected individuals were those with a positive SARS-CoV-2 virological test but without symptoms consistent with COVID-19^22^. All the recruited workers were clinically evaluated one week after the molecular test negativization (T0).

The workers recruited in the study fulfilled the roles of professors, technical and administrative clerks. Professors dealt with scientific research and teaching, technical clerks performed technical support activities for scientific research, such as informatics or laboratory activities, while administrative clerks provided front office activities for professors and students, and supported teaching activities. Exclusion criteria were moderate or severe forms of COVID-19, according to the WHO clinical progression scale^22^; any specific treatment for COVID-19, including steroids or any invasive ventilation; previous SARS-CoV-2 infection before that for which the worker was enrolled; lack of full COVID-19 vaccination; psychiatric or neurologic comorbidity at the time of sample collection.

Ten months later (T1), workers recruited in the first phase of the study were re-contacted for a follow-up evaluation, and those reporting persisting symptoms were invited to be reassessed. Recruited subjects underwent a clinical examination and repeated haematochemical tests performed in the first phase of the study. Moreover, serum samples from age and sex-matched healthy controls (HCs) were collected by the University of Siena. They had no history of autoimmune, psychiatric, or neurologic diseases, and alcohol abuse (more than 14 alcoholic units per week).

During the study, the principles of Good Clinical Practice of the International Conference on Harmonization (ICH), the 'Declaration of Helsinki' and national and international ethical guidelines were strictly followed. The Independent Ethical Committee Policlinico di Bari Hospital approved the study (protocol code 6663-Bari and protocol code 20493—Siena), and all patients signed the informed consent form.

### Biochemical assays

Peripheral blood was placed in vacutainers without additives containing separating gel and kept at room temperature for 30 min to coagulate, then centrifuged at 1600 rpm for 10 min at 4 °C. The tubes were then left standing for 1 h, after which the serum was aliquoted and stored at −70 °C before biochemical testing.

sNfL and sGFAP concentrations were assessed in each COVID-19 patient’s, both at T0 and T1, and HCs’ sample, using the commercially available immunoassay kits for NfL and GFAP-SimoaTM assay Neurology 2-Plex B (GFAP, NfL) Assay Kit (Catalog #103520; Quanterix, Billerica, MA, USA) run on the semi-automated ultrasensitive SR-X Biomarker Detection System (Quanterix). Samples were diluted 1:4 and randomly distributed on 96-well plates. The quality control (QC) samples provided with the kit had concentrations in the predefined range and the coefficient of variance between plates was < 10%. All samples were analyzed in blinded mode with alphanumeric codes. The diagnostic codes were only interrupted after the NfL and GFAP concentrations verified by the QC were reported to the database manager. The analyses were carried out at the laboratory of the Centre for Precision Medicine and Translation of the University of Siena, Italy.

For COVID-19 patients, blood samples were also collected at T0 and T1 for routine laboratory tests, on the same day as the questionnaires were administered. The analyses included hemoglobin (Hb), white blood cell (WBC), red blood cell (RBC), and platelet counts, using an automatic hematology analyser (Sysmex XE-2100, Sysmex Corporation, Kobe, Japan). Photometric method (Roche Modular, Roche Diagnostics, Mannheim, Germany) was used for measuring serum creatinine, while enzymatic tests (Sentinel Diagnostics, Milano, Italy) were used for serum AST and ALT analysis.

### Demographics, clinical features, and questionnaires

At T0, COVID-19 patients were interviewed one-on-one by trained physicians and invited to fill out a general questionnaire which included data on the patients' personal and demographic characteristics and habits (age, gender, education, cigarette smoking, alcohol consumption), their self-reported symptoms throughout the acute phase of COVID-19, the duration of these symptoms, COVID-19 treatment such as corticosteroids, intravenous immunoglobulins, antibiotics and antivirals, the number and type of their comorbidities and their regular use of medication, their vaccination status for COVID-19. Weekly alcohol units were defined using the CDC alcohol calculator^23^. An anamnestic questionnaire to assess the frequency and nature of persistent symptoms following SARS-CoV-2 infection was administered to all recruited workers at T1.

At T0, COVID-19 patients were asked to complete three further questionnaires validated for the Italian population: the Post COVID-19 Functional Status (PCFS) Scale, the Patient Health Questionnaire-9 (PHQ-9), and the Cognitive Failure Questionnaire (CFQ)^24–26^. COVID-19 patients that decided to be reassessed at T1 were asked to complete the CFQ.

The PCFS scale addresses the whole range of functional domains, in particular limitations in usual tasks or activities both at home and at work, as well as lifestyle changes. The PCFS showed, as a result, an ordinal scale of increasing severity, from grade 0 to grade 4, assessing the full range of functional limitations to capture the heterogeneity of post-COVID-19 outcomes: Death is encoded as 'D'; grade 0 means the absence of any residual symptoms; if one or more of these residual symptoms occur but do not affect the patient's usual activities, grade 1 is given; if these activities are limited in terms of intensity/frequency or occasionally avoided, grade 2 is given; grade 3 means limitations that oblige the patient to reprogram habitual activities, thus reflecting the inability to carry out some of them, which must be performed by others; grade 4, the most severe, is for severe functional limitations that require continuous assistance in everyday activities^24^.

The PHQ-9, a shorter version of the complete PHQ, is a nine-item self-report scale designed to assess depression. The 9 items correspond to the nine criteria for defining a major depressive episode according to the Diagnostic and Statistical Manual of Mental Disorders (DSM). The item answer options range from "not at all" (score 0) to "almost every day" (score 3), thus reflecting the frequency with which each symptom has bothered the interviewee in the last 2 weeks^24^. The total PHQ-9 score ranges from 5 to 9 (mild), 10–14 (moderate), 15–19 (moderately severe), and ≥ 20 (severe depressive symptoms)^25^.

Broadbent's CFQ is a self-reported assessment of failures in perception, memory, and motor function, and the score in a healthy working population is a measure of a stable cognitive resource that is involved in attention, memory, and action in daily life^26^. The questionnaire’s subscales include forgetfulness, distractibility, and false triggering. It consists of 25 questions with five responses ranging from 0 to 4, with a maximum total score of 100. The higher the score on the CFQ, the higher it is the self-perceived cognitive impairment in daily life. A CFQ total score higher than 43 has been used as indicative of significant cognitive complaints^27,28^. The questionnaire was previously used to evaluate cognitive failure in the workplace during the COVID-19 pandemic^17^.

### Statistical analysis

Data were summarised as frequency and percentage for continuous variables or as a median and interquartile range for continuous variables. To assess the normality of the distribution, the Kolmogorov–Smirnov test was performed. Since the values of sNfL and sGFAP were skewed, their levels were log-transformed. The group differences for normally distributed data were assessed using analysis of variance and Fisher's exact test. To examine sNfL and sGFAP differences between groups, analysis of covariance was performed by analyzing log sNfL and log sGFAP levels as dependent variables, COVID-19 and HC patient groups as fixed variables, and age and BMI as covariates. To confirm our results, we also performed the analysis by using the z-scores calculated by using the online application provided by Benkert et al.^29^ based on a reference database of 4532 persons with the application available at http://shiny.dkfbasel.ch/baselnflreference. The analysis of sNfL z-scores was performed with analysis of variance (ANOVA) and Tukey test. Correlation analysis was performed using two-tailed Spearman correlation coefficients. Median biomarker values at T0 and T1 were compared using a paired t-test. z-scores of the sNfL values at T0 and T1 were analysed using ANCOVA for repeated measures. A value of p < 0.05 was considered significant. Analysis of results and graphs were generated with SPSS statistics (IBM SPSS V.29, Chicago, Illinois).

### Declarations

The Local Ethics Committees approved the study (protocol code 6663-Bari and protocol code 20493-Siena), and all patients signed the informed consent form.

## Results

A group of 147 COVID-19 patients were consecutively recruited during the study period. Their demographic features, comorbidities, biochemical parameters, and scores for PCFS, PHQ-9, and CFQ are summarized in Table 1. Among them, 53 subjects (36%) were completely asymptomatic during the SARS-CoV-2 infection, while 94 (64%) showed at least one mild symptom associated with COVID-19 during the acute infection.

**Table 1:** Demographic characteristics, comorbidities, biochemical parameters and CFQ, PCFS and PHQ-9 scores in COVID-19 patients.

	COVID-19 patients		
	N (%)	Median	Range
Age (years)	147	47.0	24–66
Body mass index (kg/m^2^)	147	24.0	16–38
Sex			
Male	65 (44)		
Female	82 (56)		
Smoking habits (package/year)			
Smokers	23 (17)	10.0	2–219
Not smokers	113 (83)		
Alcohol consumption (unit/week)			
Absent	66 (45)		
Present	81 (55)	3.0	1–14
Comorbidities			
Hypertension	5 (3.4)		
Asthma	3 (2.0)		
Diabetes	1 (0.7)		
RBC (mg/dL)		4.77	3.60–6.38
WBC (mg/dL)		6.7	2.3–19.8
Hb (g/dL)		13.7	10.0–17.0
HCT (%)		40.7	32.2–50.2
MCV (fl/cell)		86	61–94
Neutrophils (%)		59	32–85
Lymphocytes (%)		30	6–52
Monocytes (%)		8	1–13
Eosinophils (%)		2	1–26
Basophils (%)		1	0–2
Platelets (mg/dL)		244	134–381
ALT (U/L)		19	10–82
AST (U/L)		17	10–40
Creatinine (mg/dL)		0.92	0.31–1.51
CFQ score			
> 43	11	50	45–53
< 43	136	16	5–42
PCFS scale			
Grade 0	76 (51.0)		
Grade 1	69 (47.0)		
Grade 2	5 (3.4)		
PHQ-9			
No symptoms	50 (34.0)		
Mild	86 (58.5)		
Moderate	11 (7.5)		

Considering the PCFQ scales, 69 subjects (46.9%) showed residual symptoms with no impact on daily life (grade 1), and only five subjects (3.4%) showed mild symptoms with negligible impact on daily life (grade 2). According to PHQ-9 results, depressive conditions were reported as mild in 86 patients (58.5%), and moderate in 11 patients (7.5%). The CFQ questionnaire showed individual cognitive failure, according to a score higher than 43, in 11 COVID-19 patients. Comparison between COVID-19 patients with CFQ > 43 vs < 43 showed no significant differences in PHQ scores (median score 13 vs 15, respectively) and no significant correlation was found between the PHQ and CFQ scores. Demographic features of HCs are summarised in Table 2.

**Table 2:** Demographic characteristics of healthy controls.

	Healthy controls		
	N (%)	Median	Range
Age (years)	82	48.5	30–64
Body mass index (kg/m^2^)	82	25	24–25
Male	30 (36)		
Female	52 (63)		

**Figure 1:**
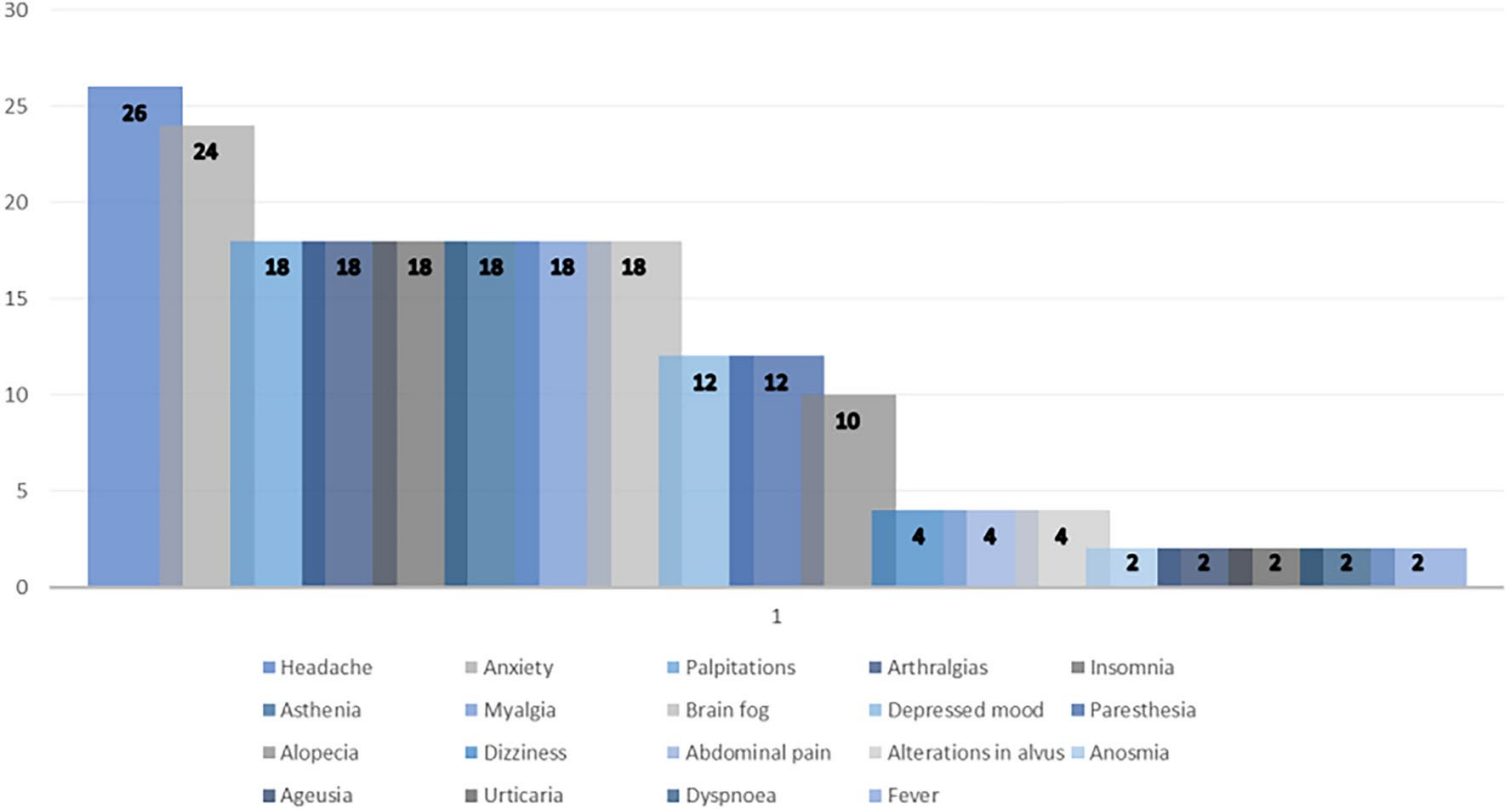
Prevalence of the symptoms reported by the COVID-19 patients at T1.

A total of 49 workers agreed to be reassessed at T1 reporting the continuation or development of new symptoms after the initial SARS-CoV-2 infection, with these symptoms lasting for at least 2 months with no other explanation . The prevalences of the reported symptoms are showed in Fig. 1, whereas demographic characteristics and haematochemical parameters at T1 are shown in Table 3. The most prevalent symptoms were headache (26%) and anxiety (24%), followed by palpitations, arthralgias and insomnia (18%).

**Table 3:** General features and haematochemical parameters of workers recruited 10 months after a previous SARS-CoV-2 infection.

Demographic features and haematochemical parameters	Post-COVID workers (N = 49)		
	N (%)	Median	Range
Age (years)		49.0	25–63
BMI (kg/m^2^)		23.0	17–30
Gender			
Man	22 (44)		
Women	28 (56)		
Smoking habit			
Smokers	10 (20)		
Not smokers	40 (80)		
Alcohol consumption	35 (70)		
Comorbidity			
Hypertension	3 (6)		
Asthma	3 (6)		
Diabetes	0 (0)		
Hepatopathies	0 (0)		
Glycemia (mg/dl)		82.5	80–108
Creatinine (mg/dl)		0.86	0.84–1.17
AST (U/l)		18.5	13–42
ALT (U/l)		20.0	10–47
Gamma-GT (U/l)		22.5	12–79
Hb (g/dl)		15.4	12.1–17.1
RBC (10^6^/mmc)		4.88	4.5–5.81
WBC (10^3^/mmc)		6.38	5.52–13.97
Platelets (10^3^/mmc)		277.5	211–420

*BMI* body mass index, *ALT* alanine transaminase, *AST* aspartate transaminase, *GGT* gamma-glutamyltransferase, *HB* hemoglobin, *RBC* red blood cells, *WBC* white blood cells.

### sNfL and sGFAP levels

**Figure 2:**
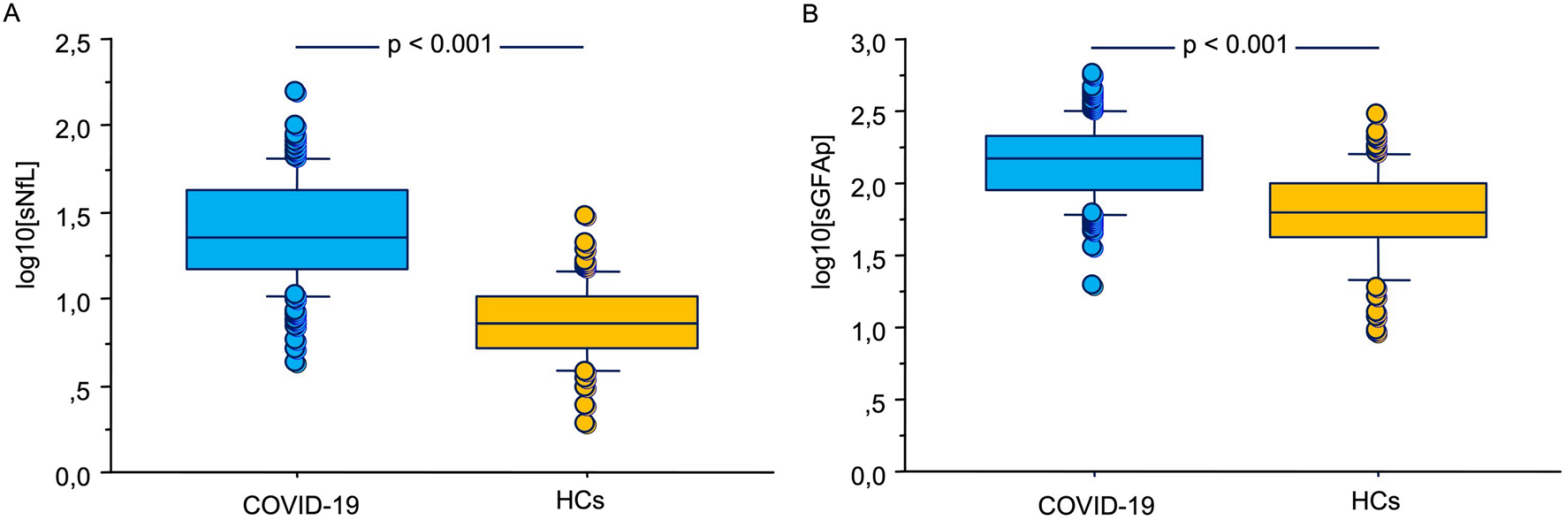
Log10 levels of serum neurofilament light chain (sNfL) (**A**), and glial fibrillary acidic protein (sGFAp) (**B**) in COVID-19 patients and healthy controls (HCs). Box plots express the first (Q1) and third (Q3) quartiles by the upper and lower horizontal lines in a rectangular box, in which there is a horizontal line showing the median. The whiskers extend upwards and downwards to the highest or lowest observation within the upper (Q3 + 1.5 × IQR) and lower (Q1 − 1.5 × IQR) limits.

**Figure 3:**
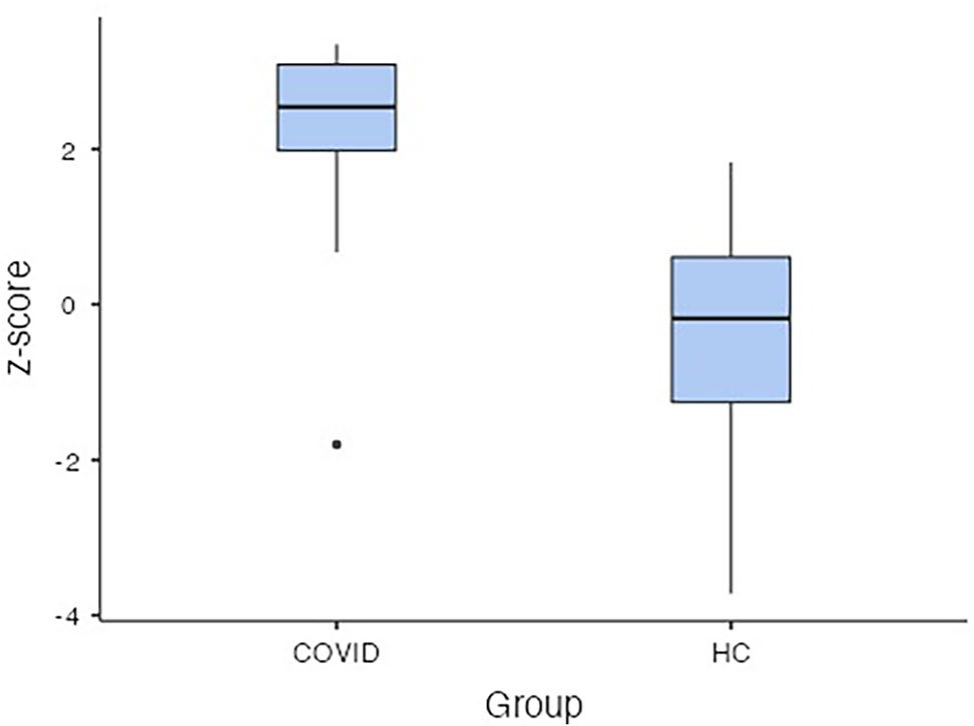
z-scores of sNfL at T0 calculated by using the online application provided by Benkert et al. (ref) (http://shiny.dkfbasel.ch/baselnflreference) showing the difference between COVID-19 patients and healthy controls (HCs). p value < 0.001. Box plots express the first (Q1) and third (Q3) quartiles by the upper and lower horizontal lines in a rectangular box, in which there is a horizontal line showing the median. The whiskers extend upwards and downwards to the highest or lowest observation within the upper (Q3 + 1.5 × IQR) and lower (Q1 − 1.5 × IQR) limits.

At T0, sNfL and sGFAP levels were significantly higher in COVID-19 patients (sNfL median 22.83 pg/ml, IQR 14.71–42.36 pg/ml; sGFAP median 146.32 pg/ml, IQR 90.08–209.53 pg/ml) than in HCs (sNfL median 7.21 pg/ml, IQR 5.17–10.30 pg/ml; sGFAP median 63.53 pg/ml, IQR 841.97–101.33 pg/ml; p < 0.001 for both) (Fig. 2). The analysis performed by using sNfL z-scores confirmed that COVID patients had increased levels of sNfL than HCs (p < 0.001, mean difference 2.73; standard error 0.217, see Fig. 3).

sNfL and sGFAP levels were significantly correlated with age, both in COVID-19 patients (sNfL: r = 0.44, sGFAP: r = 0.33; both p < 0.001) and in HCs (sNfL: r = 0.62, p < 0.001; sGFAP: r = 0.35, p = 0.002), and with each other (r = 0.47, COVID-19 patients; r = 0.81, HCs; both p < 0.001). Moreover, in COVID-19 patients, sNfL levels were correlated with the number of alcohol units consumed per week (r = 0.30, p < 0.05). No significant correlation was observed between sNfL or sGFAP levels and the duration of the SARS-CoV-2 infection, the duration of the different symptoms during the acute phase, or the other biochemical parameters analyzed. Finally, none of the post-COVID symptoms, including headache, anosmia, ageusia, nor sleep disturbance was associated with sNfL and sGFAP levels.

**Figure 4:**
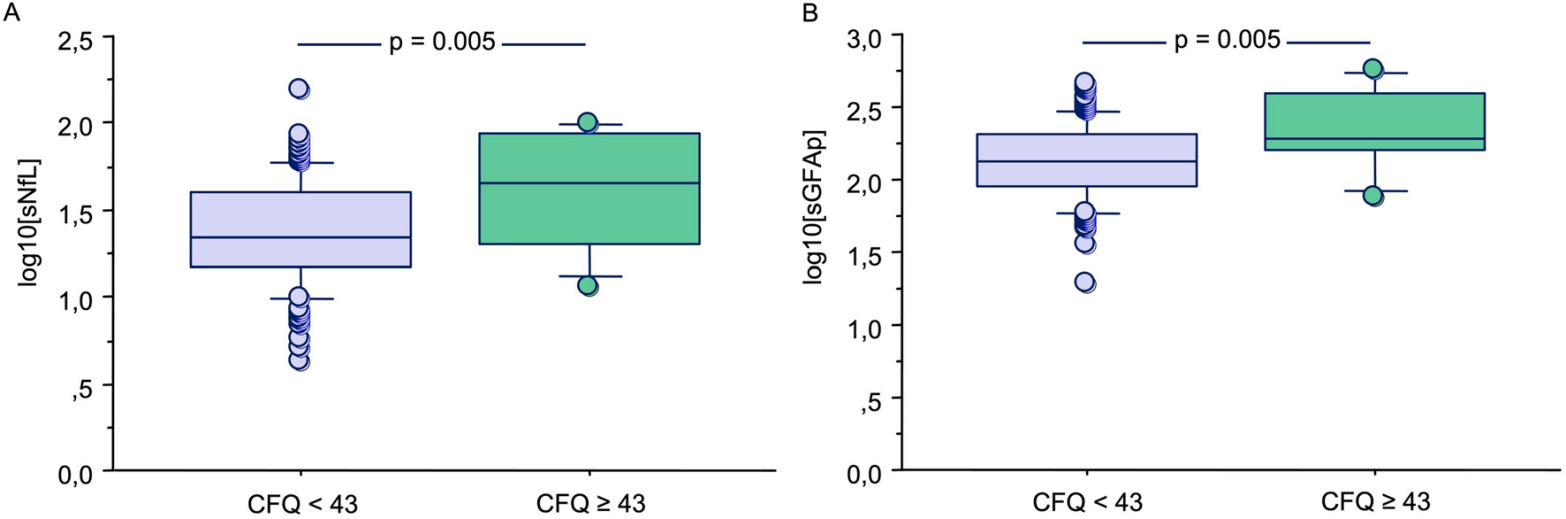
Log10 levels of serum neurofilament light chain (sNfL) (**A**), and glial fibrillary acidic protein (sGFAp) (**B**) in COVID-19 patients presenting cognitive failure (CFQ ≥ 43), and in those without cognitive failure (CFQ < 43). Box plots express the first (Q1) and third (Q3) quartiles by the upper and lower horizontal lines in a rectangular box, in which there is a horizontal line showing the median. The whiskers extend upwards and downwards to the highest or lowest observation within the upper (Q3 + 1.5 × IQR) and lower (Q1 − 1.5 × IQR) limits.

The eleven COVID-19 patients presenting a CFQ score higher than 43 (7.5% of the total sample) showed significantly higher levels of sNfL (median 45.03 pg/ml, IQR 19.97–87.31 pg/ml) and sGFAP (median 194.15 pg/ml, IQR 157.81–393.88 pg/ml) than the COVID-19 patients showing CFQ < 43 (sNfL: median 22.42 pg/ml, IQR 14.66–39.74 pg/ml; sGFAP: median 131.28 pg/ml, IQR 89.75–206.96 pg/ml; p = 0.005 for both) (Fig. 4). No significantly different levels of sNfL and sGFAP were observed according to the grade of the PCFS scale and PHQ-9 groups. Finally, no significant correlations were found between scores obtained with the PHQ-9 or CFQ questionnaires and sNfL and sGFAP levels.

**Figure 5:**
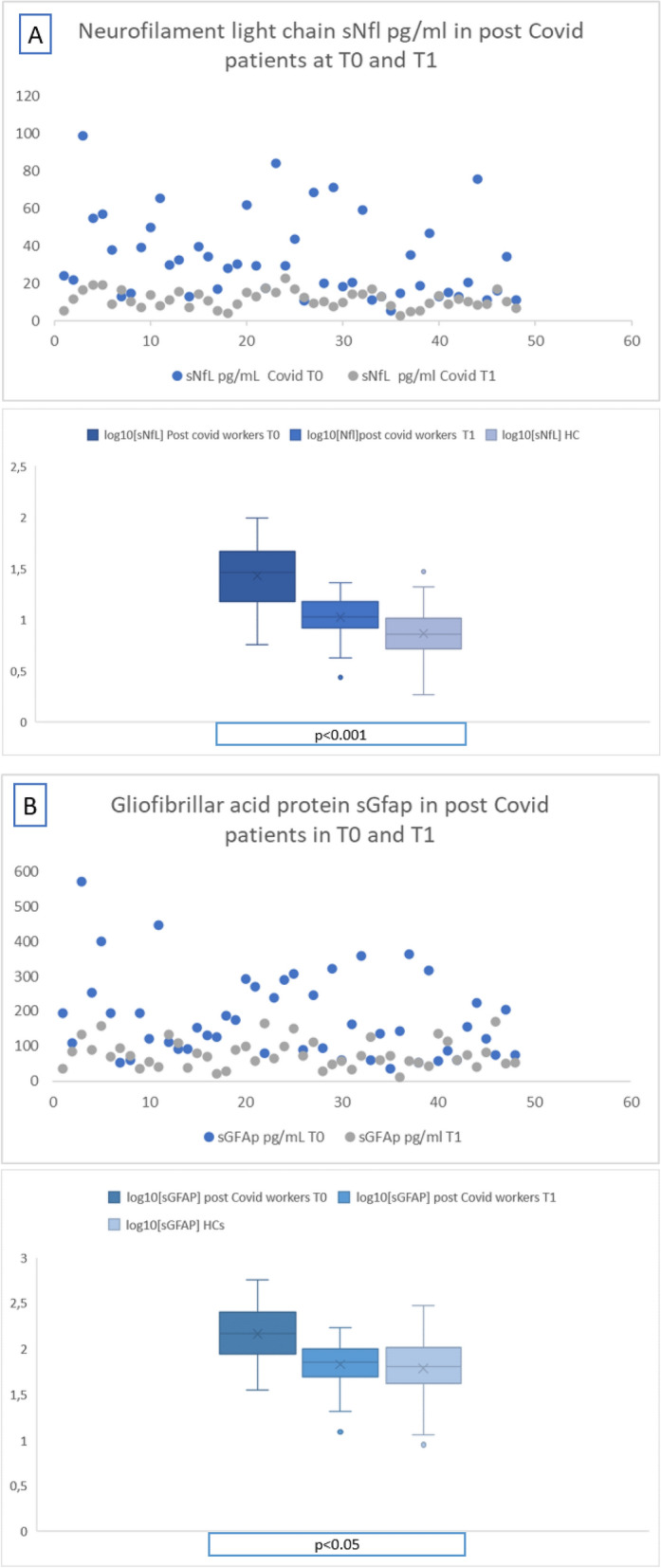
Longitudinal trend and median levels of sNfL (**A**) and sGFAP (**B**) among the COVID-19 patients, analysed at T0 and T1, and the the healthy controls.

**Figure 6:**
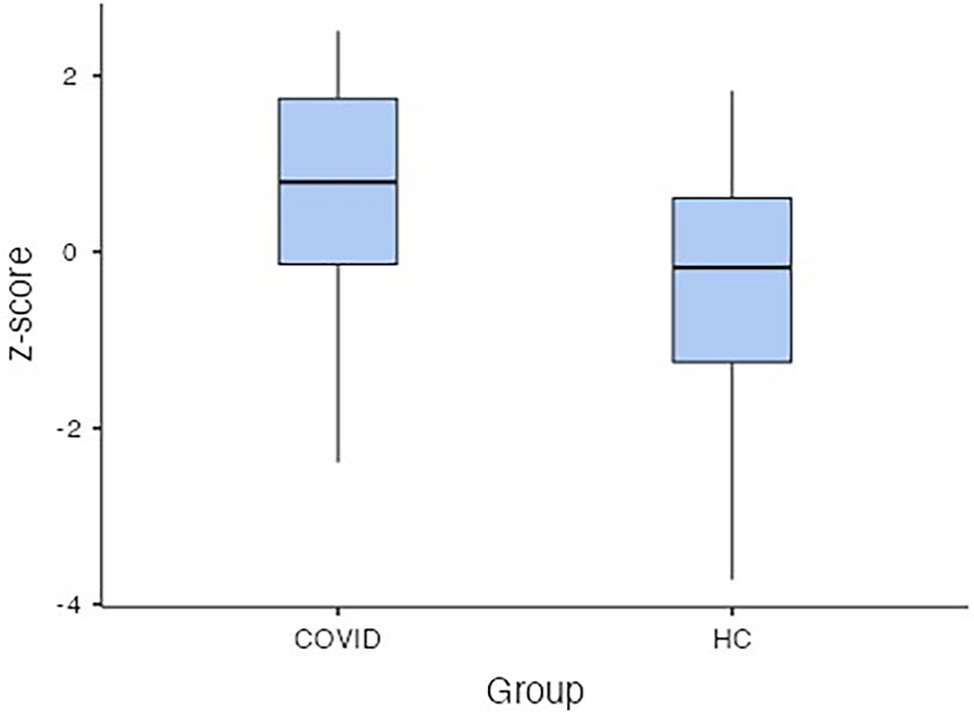
z-scores of sNfL at T1 calculated by using the online application provided by Benkert et al. (ref) (http://shiny.dkfbasel.ch/baselnflreference) showing the difference between COVID-19 patients and healthy controls (HCs). p value < 0.001. Box plots express the first (Q1) and third (Q3) quartiles by the upper and lower horizontal lines in a rectangular box, in which there is a horizontal line showing the median. The whiskers extend upwards and downwards to the highest or lowest observation within the upper (Q3 + 1.5 × IQR) and lower (Q1 − 1.5 × IQR) limits.

At the subsequent follow-up (T1), sNfL and sGFAP levels in the 49 COVID-19 patients, showed a significant reduction of (median sNfL 18.3 pg/mL; median sGFAP 77.2 pg/mL) if compared to the first assessment (Fig. 5), but a significant increase if compared to the control group (median sNfl 7.2 pg/mL, median sGFAP 63.5 pg/mL). The analysis performed by using sNfL z-scores at T1 confirmed that COVID patients still had increased levels of sNfL than HCs (p < 0.001, mean difference 1.02; standard error 0.232, see Fig. 6). The ANCOVA for repeated measures showed that the reduction of z-scores values was significant (p < 0.001).

At T1, paired T test showed that COVID-19 patients also had significantly higher mean CFQ values than their values at T0 (18.1 vs. 27.1; p < 0.01). Seven of the 49 COVID-19 patients (14.3%) presented CFQ score higher than 43, although sNfL (9.72 pg/ml) and sGFAP (64.72) levels did not differ significantly if compared to those with CFQ < 43 (sNfL 11.47 pg/ml ; sGFAP 76.94 pg/ml).

## Discussion

Our study shows that the increase in serum biomarkers of neuronal and glial damage, sNfL and sGFAP, was present one week after resolution of asymptomatic SARS-CoV-2 infection or mild COVID-19 and was more pronounced in patients with cognitive impairment. Furthermore, 10 months after resolution of the infection, levels of these biomarkers were still significantly higher than in healthy controls, although reduced from those observed at baseline. At the same time, self-reported cognitive impairment appeared to worsen in the same subjects, suggesting that early neuronal and glial damage may have resolved by 10 months post-infection, although subjective cognitive impairment may persist or become more pronounced.

sNfL and sGFAP half-lives should be considered when interpreting the results. It is well known that sNfL concentrations increase with a maximum between 7 and 10 days following a nervous system injury and have a half-life of up to a month or two^30–32^. sGFAP concentrations, instead, increase within 1 h following a CNS injury and then peaks within 20–24 h, with a following decline over 72 h, having a biological half-life of 24–48 h^33–35^. A possibly ongoing damage involving neurons and astrocytes can be hypothesized after SARS-Cov2 negativization. Our study seems to be in line with previous reports showing reduced levels of biomarkers of neuronal damage after three and six months after acute COVID-19 infection^36–41^ However, unlike these studies, our data show higher levels of these biomarkers 10 months after resolution of the infection in subjects with persistent symptoms compared with HCs. The pathological and neurobiological mechanisms responsible for these delayed increases in biomarkers remain uncertain. However, prior investigations have demonstrated a correlation between the sustained elevation of these biomarkers for months following an acute central nervous system (CNS) injury and diffusion tensor imaging (DTI) metrics indicative of microstructural damage in both grey and white matter. This correlation suggests that these biomarkers may signify distinct aspects of ongoing axonal pathology, with neurofilament light chain (NFL) potentially indicating ongoing axonal loss, while glial fibrillary acidic protein (GFAP) reflects glial responses to this evolving damage^39,42^.

The second relevant finding of our study is represented by the higher sNfL and sGFAP levels in the eleven COVID-19 patients complaining of cognitive failures at T0. Cognitive deficits are common after COVID-19 and can impair executive functions, attention, and episodic memory^40,41,43,44^. Studies on the neuropsychological alterations during acute COVID-19 and in the post-COVID-19 phase show inhomogeneous results, particularly for the variable time of the evaluation, ranging from two to five weeks after the onset, up to one year after the recovery^42,43,45,46^. Most of these studies, however, mainly focus on hospitalized patients, being non-hospitalised patients somehow overlooked. A recent study showed that more than one-third of hospitalized and non-hospitalized patients after COVID-19 experienced a perceived cognitive deficit after 30 days after hospitalization or outpatient infection. It should be noticed that, differently from our population, this patient cohort included mainly hospitalized patients with remarkable comorbidities^43,46^. Our study shows cognitive failure immediately following the recovery in a not negligible percentage (7.5%) of COVID-19 patients, suggesting a clinical impact of SARS-CoV-2 even in individuals with the mildest forms of the disease. The results of the PHS questionnaire and the concurrent increase of sNfL and sGFAP clearly indicate that the cognitive failure was associated with CNS damage, and not with a depressive mood. No clear association was observed between CFR > 43 (14.5% of the total sample) and sNfL and sGFAP levels at T1. The greater number of COVID-19 patients with a higher CFQ score at ten months may be multifactorial, reflecting cumulative neuronal and astroglial damage in the central nervous system^39,42^.

The previous literature on mild or non-severe COVID-19 cases clearly indicates their significant impact on cognitive function, particularly in domains such as working memory and processing speed^44,47^, with a good potential for recovery over time though some impairments may persist^45,46,48,49^. Cognitive failures, therefore, may interfere with highly complex working activities, particularly those that require attending to and remembering large amounts of information, like academic or administrative jobs. Our patients with CFQ scores higher than 43 could experience high difficulties when returning to their work. The negative impact on daily functioning and quality of life of post-COVID cognitive dysfunction has been highlighted by Quan et al.^47,50^ emphasizing the economic, health, and social burden associated with. In fact, Beck and Flow^48,51^ demonstrate that individuals who had contracted SARS-CoV-2 infection reported cognitive failures at work and difficulty performing their tasks, highly detrimental to their performance, and may leave a job looking for other sources of employment. This evidence provides support for the need to perform careful neuropsychological evaluations for all the workers following SARS-CoV-2 infection, to allow both an adequate resumption of work activities and to monitor the onset of any cognitive impairment even in workers with a previous mild COVID-19 or asymptomatic infection. In particular, the assessment of mild cognitive impairments may allow the implementation of specific preventive strategies aimed at improving the psychological well-being of these workers^49,52^.

Further studies are needed to assess the extent to which job-specific characteristics may influence the degree of potential correlation between cognitive failure and COVID-19. For example, it is well documented that the strength of the relationship between general mental ability and job performance is moderated by job complexity, suggesting that a positive relationship between general mental ability and job performance is stronger for highly complex jobs than for low-complexity jobs^50,53^. Previous studies, moreover, demonstrated a positive correlation between accident occurrence and individual cognitive failure, which should be considered as one of the reasons for increasing unsafe behaviors^51,52,54,55^. In this light, the importance of evaluating cognitive failure in occupational settings is also strictly related to the associated increased risk of accidents at work.

Finally, we described a positive association between alcohol consumption and sNfL and sGFAP levels in COVID-19 patients. A past medical history of excessive and chronic alcohol consumption has already been proposed as a risk factor for severe COVID-19^53,56^. Interestingly, a significant and similar upregulation of a few genes associated with both severe COVID-19 and chronic alcoholism has been demonstrated, suggesting that chronic alcoholism represents an important risk factor for brain injury in COVID-19 patients^54,55,57,58^. Moreover, previous reports demonstrate increased sNfL levels in subjects with alcohol use disorders, which may be useful as an early serological indicator of alcohol-induced brain injury^56,57,59,60^. Chronic alcohol consumption has been linked to a compensatory upregulation of ACE2 in the brain, a phenomenon attributed to disturbances in the Renin-Angiotensin System (RAS)^58,61^. This alcohol-induced ACE2 upregulation may increase the risk or severity of SARS-CoV-2 infection^59,62^. Additionally, chronic alcohol intake has been associated with the regulation of ACE2 gene expression, potentially influencing the susceptibility to SARS-CoV-2 infection^60,63^. Our data emphasize the importance of evaluating alcohol consumption, also considering the negative effects on cognitive performance even at low doses^61,62,64,65^.

The main limitation of our study was the use of Broadbent's CFQ which is a self-reported assessment of cognitive performance and is far from diagnosing an objective neurocognitive impairment. It can be hypothesized that many of the subjects who reported an impair in CFQ may have performed normally in the standardized neuropsychological tests and function normally in daily routine. Nonetheless, the CFQ questionnaire represents a reliable measure of failures of human performance under real-life conditions and is a good indicator of cognitive control functioning^63,64,66,67^. This questionnaire, therefore, can be regarded as a good tool to explore cognitive deficits impairing working activity and has already been used in similar contexts^65,68^. A further limitation of the study was the impossibility of performing a correlation analysis between alcohol consumption and serum neurofilament levels in HCs. Finally, we fully acknowledge that it is not possible to distinguish the origin of NfL between the peripheral and central nervous systems. While the majority of the NfL signal in blood originates from the central nervous system^66,69^, NfL is also expressed in the peripheral nervous system and has been used as a biomarker for peripheral neuropathy. Therefore, a significant involvement of the peripheral nervous system in the process of neuronal damage cannot be excluded. However, it is worth noting that none of our patients reported any signs or symptoms of peripheral neuropathy.

## Conclusions

In summary, our findings indicate a potential ongoing injury affecting neurons and astrocytes following SARS-CoV-2 negativization, evident ten months after negativization. Moreover, this phenomenon appears to be more pronounced in individuals experiencing cognitive impairment one week post-SARS-CoV-2 infection. Further studies are needed to better characterize the long-term trend of sNfL and sGFAP elevation and to explore the long-term consequences of CNS damage in these patients, particularly in performing their job activities. This could be useful in the view of adopting prevention measures to improve worker health after a mild COVID-19 or an asymptomatic SARS-CoV-2 infection.

## Data Availability

The datasets used and/or analyzed during the current study are available from the corresponding author upon reasonable request.
